# Sexual health among the oldest old: a population-based study among people aged 85 years and older in Stockholm, Sweden

**DOI:** 10.1093/sexmed/qfae022

**Published:** 2024-04-05

**Authors:** Marios Politis, Kyriaki Kosidou, Stefan Fors, Martina Nord

**Affiliations:** Centre for Epidemiology and Community Medicine, Region Stockholm, Stockholm SE-104 31, Sweden; Centre for Epidemiology and Community Medicine, Region Stockholm, Stockholm SE-104 31, Sweden; Department of Global Public Health, Karolinska Institutet, Stockholm 171 77, Sweden; Centre for Epidemiology and Community Medicine, Region Stockholm, Stockholm SE-104 31, Sweden; Aging Research Center, Karolinska Institutet & Stockholm University, Stockholm SE-113 30, Sweden; Department of Public Health Sciences, Stockholm University, Stockholm 114 19, Sweden; Centre for Epidemiology and Community Medicine, Region Stockholm, Stockholm SE-104 31, Sweden; Department of Global Public Health, Karolinska Institutet, Stockholm 171 77, Sweden

**Keywords:** sexual health, older adults, oldest old, healthy ageing

## Abstract

**Background:**

Sexual health is an important contributor to the well-being and life satisfaction of people aged ≥85 years, known as the *oldest old*. However, little is known about sexual health in this population.

**Aim:**

To examine aspects of sexual health among the oldest old and explore its associations with sociodemographic, health-related, and lifestyle factors.

**Methods:**

We conducted a population-based cross-sectional study including 183 individuals aged ≥85 years who were residents in Stockholm County, Sweden. Responders (response rate, 63%) were interviewed on a range of health, sociodemographic, and lifestyle parameters, including aspects of sexual health. Participants’ responses to the sexual health items were reported as proportions with 95% CIs. Associations were examined with multivariable logistic regression.

**Outcomes:**

We examined sexual activity, sexual satisfaction, problems related to sexual health, and inquiries on sexual health by a health care provider.

**Results:**

Twelve percent of participants (95% CI, 8%-17.6%) were sexually active, and 63.9% (95% CI, 56.5%-70.9%) were satisfied with their sexual lives during the past year. A third (35%; 95% CI, 28.4%-42.2%) reported at least a problem related to sexual health. Only 2.2% (95% CI, 0.6%-5.5%) were asked about sexual health by a health care provider, while 8.2% (95% CI, 4.7%-13.2%) identified a need for such an assessment. Yet, 85.2% (95% CI, 79.3%-90.0%) indicated no need for their sexual health to be evaluated by a health care provider. Being partnered was positively associated with sexual activity (adjusted odds ratio, 9.13; 95% CI, 2.53-32.90), whereas having strong social support was positively associated with being satisfied with one’s sexual life (adjusted odds ratio, 2.96; 95% CI, 1.53-5.74).

**Clinical Implications:**

Health care providers should be proactive in assessing the sexual health of the oldest individuals.

**Strengths and Limitations:**

A representative sample of an underresearched population was used in this study. However, the generalizability of our findings may be restricted due to the small sample. To maintain statistical power from a relatively small sample, we might have lost explanatory power. Given the observational cross-sectional nature of the data, we cannot draw causal inferences based on the observed associations.

**Conclusions:**

A 10th of participants were sexually active, and the majority were satisfied with their sexual lives. Although many participants reported problems related to sexual health, few expressed the need to discuss sexual health with health care providers. Future studies should explore potential barriers to addressing sexual health and unmet health care needs among the oldest old.

## Introduction

Globally, 1 in 6 people will be older than 65 years by 2050, and the proportion of individuals aged ≥80 years is projected to experience the most significant growth of all age groups, reaching >10% of the total population in many countries by this year.^1^^,^^2^ Although an unprecedented human success, increased life expectancy comes with many challenges: exclusion and discrimination of older people in work and social life, uncertain social protection, heavy disease load, and barriers to accessing health care and ensuring a healthy life.^3^ Unmet needs in health care and social support among older adults have been identified in several countries, particularly among those aged >85 years, often referred to as the *oldest old*.^4–7^

A nonnegligible proportion of older adults remains sexually active even into their 9th and 10th decades of life, expressing a variety of physical and emotional intimacy.^8^^,^^9^ Sexual activity in later life contributes to overall well-being and life satisfaction and has been recognized as a crucial aspect of healthy aging, with older people often expressing satisfaction with sex life.^10–18^ However, older individuals are often perceived as sexually inactive or asexual, lacking space to express their sexuality, as well as access to essential sexual health services, while sexual health in later life is often overlooked by health care providers (HCPs), researchers, and policy makers.^9^^,^^11^^,^^12^^,^^19–24^

Chronic physical and mental health problems may pose difficulties on sexual functioning and reduce the sexual activity and satisfaction of older people.^11^^,^^15^^,^^18^^,^^25–28^ Common sexual problems in older men and women include a lack of sexual drive and orgasmic difficulties.^25^^,^^26^ Older men often have erectile dysfunction and testosterone deficiency, while women mainly experience vaginal dryness and pain during sex.^27^^,^^28^ Furthermore, socioeconomic factors and psychological stress may affect older adults’ sexual lives, such as limited privacy, difficulties in discussing sexual health problems with an HCP, and stress due to caregiving or managing a chronic illness.^11^

This study capitalized on recently collected data from a population-based survey among the oldest old residents of Stockholm County, Sweden, to report on the sexual health of the oldest old. Our research questions were as follows:

• What is the prevalence of sexual activity, sexual satisfaction, and problems related to sexual health among the oldest old?

• What are the associations of sociodemographic, health-related, and lifestyle factors among the oldest old with sexual health?

• How many among the oldest old have received inquiries on sexual health by an HCP, and how many report an unmet need for such inquiries?

## Methods

### SWEOLD study

We used data from the latest wave of SWEOLD (2021): the Swedish Panel Study of Living Conditions of the Oldest Old, an ongoing national representative study of the older population of Sweden.^29^ Data on the sexual health of the oldest old were collected for the first time for the subsample residing in Stockholm County.^30^

### Participants

Our study sample comprised a random sample of 500 Stockholm County residents aged ≥85 years who were invited to participate after being identified through Statistics Sweden via the unique Swedish personal identification numbers assigned to all citizens. Out of these, 63 were deceased prior to the interview and 160 chose not to participate, yielding a sample of 277 community-dwelling and care facility residents (63% response rate). Of those, 73 participated in the study through indirect interviews (ie, through relatives or caregivers) and therefore were excluded from the sexual health section due to frailty or cognitive impairment. After further exclusion of 21 people who, though directly interviewed, chose not to respond to the sexual health section, our analytic sample finally included 183 participants. All interviews were administered via telephone between fall 2021 and spring 2022.

### Ethical approval

All study participants provided informed consent, and the study received approval from the Swedish Ethical Review Authority (Dnr 2021-00393).

### Main analysis

#### Sexual health assessment

Sexual activity during the past 12 months was assessed with the following question: “Have you been sexually active during the past 12 months?” with *yes*, *no*, or *do not know* as possible replies*.* The following clarification of what is meant by “sexual activity” was provided by the interviewer to the study participants: “By sex is meant not only penetration but also caressing, touching, oral sex, masturbation, or other forms of intimacy. It can be with yourself or with a partner.” Satisfaction with sexual life during the past 12 months was examined by asking, “How have you experienced your sexual life during the past 12 months?” with 3 alternatives: *mostly satisfied*, *mostly dissatisfied*, or *do not know.* Responses to the items on sexual activity and sexual satisfaction were dichotomized by collapsing the *no* and *do not know* answers into 1 *no/do not know* category. We evaluated whether individuals had encountered any problems related to sexual health in the past year by asking, “Have you faced any of these problems in the last 12 months?” to which responders could choose none, 1, or more of the following options: lack of sexual desire, lack of pleasure during sex, lack of a sexual partner, difficulty finding a private place for sex, physical health problems affecting sexual life, and anxiety or worry affecting sexual life. We combined responses to these items by coding *any problem related to sexual health.* Those who reported experiencing at least 1 of the listed problems were categorized as *yes*, while those who indicated none of these problems were categorized as *no*. Inquiries regarding sexual health by HCPs were examined with the question “Have you been asked about sexual health or sex life issues when you came into contact with HCPs during the past 5 years?” with 4 possible answers: *yes*; *no, but I did not need it*; *no, but I needed it*; and *do not know.*

#### Other covariates

Other self-reported covariates were as follows. Partnered individuals were defined as those married or being in romantic relationships. Participants’ educational level was dichotomized into having a university degree or not. Having strong social support (yes/no) was defined as having someone to ask for support in all the following situations: in case of illness; in need for socializing; in need of expressing personal concerns; in need of borrowing SEK 16000 (approximately €1385 or $1540); and in need of choosing, changing, or complaining to, for example, health care services or electricity company. Participants were also asked to define their self-perceived health as good, bad, or somewhere in the middle. Self-perceived health was dichotomized into 2 categories, good and fair to poor, by collapsing the 2 last answer alternatives. The 4-item Geriatric Depression Scale (GDS-4) was used to define a binary variable on risk for depression.^31^ Respondents who scored 0 or 1 point in the GDS-4 were categorized as having a low risk for depression, whereas those scoring 2 to 4 points were categorized as having a high risk for depression. Alcohol consumption was categorized as medium to high or medium to low, with the cutoff set to drinking wine, beer, or alcohol spirits equal to or more than 1 or 2 times per week. Smoking history was dichotomized into having smoked, including those currently smoking or having previously smoked, and never smoked.

### Statistical analysis

Stata version BE 17 (StataCorp) was used for all analyses in the current study. Participants’ characteristics are presented as numbers and percentages. Chi-square and Mann-Whitney *U* tests were used to identify significant sex differences at a significance level of 95%. Percentages with corresponding 95% CIs were calculated to investigate the distribution of responses to the 4 sexual health items with the Clopper-Pearson exact method. Multivariable logistic regression analysis was used to examine the relationship of sociodemographic, health-related, and lifestyle characteristics with sexual health. To assess more precisely the association of participants’ characteristics with the sexual health items, all regression models were adjusted for all other examined characteristics and presented as adjusted odds ratios (aORs) with 95% CIs.

### Post hoc analysis

We conducted a post hoc analysis to explore the observed discrepancy between participants’ low sexual activity and relatively high sexual satisfaction. First, we investigated the association between sexual activity (exposure) and sexual satisfaction (outcome) with binary logistic regression, adjusted for participants’ characteristics. We also combined responses to the sexual activity and sexual satisfaction items to create a new multinomial variable with 3 categories:

Sexually nonsatisfied: participants responding *no* or *do not know* when asked about being sexually satisfied

Sexually satisfied and sexually nonactive: participants who reported being sexually satisfied and *no* or *do not know* to the question about sexual activity

Sexually satisfied and sexually active: those who responded *yes* to the sexual satisfaction and sexual activity items

We examined the association between this polytomous outcome and participants’ characteristics with adjusted multinomial logistic regression.

## Results

### Characteristics of study participants

The median age of the participants was 88 years (IQR, 86-90) and 32.8% were older than 90 years (Table 1). One-third had university education and 61.2% reported strong social support. More men than women were partnered (*P* < .001) and past or active smokers (*P* = .025). About 40% reported good self-perceived health and medium to high alcohol consumption. Last, 22% were estimated to have a high risk for depression according to the GDS-4.

**Table 1:** Characteristics of the participants: overall and by sex.^a^

**Characteristic**	**Total (*N* = 183)**	**Men (*n* = 64)**	**Women (*n* = 119)**	***P* value** ^**b**^
Age, y, median (IQR)	88 (86-90)	87 (86-91)	88 (86-90)	.33
Age group, y				.75
85-89	123 (67.2)	44 (68.8)	79 (66.4)	
≥90	60 (32.8)	20 (31.2)	40 (33.6)	
University degree				.33
Yes	63 (34.3)	25 (39.1)	38 (31.9)	
No	120 (65.7)	39 (60.9)	81 (68.1)	
Partnership status				<.001
Partnered	68 (37.2)	40 (62.5)	28 (23.5)	
Not partnered	115 (62.8)	24 (37.5)	91 (76.5)	
Strong social support				.49
Yes	112 (61.2)	37 (57.8)	75 (63.0)	
No	71 (38.8)	27 (42.2)	44 (37.0)	
Self-perceived health				.63
Good	73 (39.9)	24 (37.5)	49 (41.2)	
Fair to poor	110 (60.1)	40 (62.5)	70 (58.8)	
Risk of depression (GDS-4)				.06
Low	40 (21.9)	9 (14.1)	31 (26.1)	
High	143 (78.1)	55 (85.9)	88 (73.9)	
Alcohol consumption				.11
Medium to high	74 (40.4)	31 (48.4)	43 (36.1)	
Medium to low	109 (59.6)	33 (51.6)	76 (63.9)	
Smoking history				.025
Smoked	74 (40.4)	33 (48.4)	41 (34.5)	
Never smoked	109 (59.6)	31 (51.6)	78 (65.5)	

Abbreviation: GDS-4, 4-item Geriatric Depression Scale.

^a^Data are presented as No. (%) unless noted otherwise.

^b^Chi-square (all cells have expected counts ≥3) and Mann-Whitney *U* tests assess differences in characteristics between men and women at a significance level of 95%.

### Sexual health of the oldest old

About a 10th of the participants (12%; 95% CI, 8%-17.6%) were sexually active during the past year: 8.4% (95% CI, 4.1%-14.9%) among women and 18.7% (95% CI, 10.1%-30.5%) among men (Table 2). Most (63.9%; 95% CI, 56.7%-70.6%) were satisfied with their sexual lives, with small differences between sexes. About a third (35%; 95% CI, 28.4%-42.2%) reported at least 1 problem related to sexual health: 28.6% (95% CI, 21.1%-37.4%) among women and 46.9% (95% CI, 40.7%-65.2%) among men. Only 2.2% (95% CI, 0.8%-5.7%) had received a question on sexual health by an HCP. Yet, most (85.2%; 95% CI, 79.3%-90.0%) did not feel the need for an inquiry. However, 8.2% (95% CI, 4.7%-13.2%) expressed such a need: 12.5% (95% CI, 5.6-23.2%) among men and 5.9% (95% CI, 2.4%-11.7%) among women.

**Table 2:** Sexual health among the oldest old (≥85 years) in Stockholm County.

	**% (95% CI)** ^**a**^		
**Characteristic**	**Total (*N* = 183)**	**Men (*n* = 64)**	**Women (*n* = 119)**
Sexually active in the past 12 mo			
Yes	12.0 (8.0-17.6)	18.7 (10.1-30.5)	8.4 (4.1-14.9)
No	83.6 (77.4-88.7)	76.6 (64.3-86.2)	87.4 (80.1-92.8)
Do not know	4.4 (1.9-8.4)	4.7 (1.0-13.1)	4.2 (1.4-9.5)
Sexually satisfied in the past 12 mo			
Mostly satisfied	63.9 (56.5-70.9)	59.4 (46.4-71.5)	66.4 (57.2-74.8)
Mostly dissatisfied	5.4 (2.7-9.8)	9.4 (3.5-19.3)	3.4 (0.9-8.4)
Do not know	30.6 (24.0-37.8)	31.2 (20.2-44.1)	30.2 (22.2-39.3)
Having any problem related to sexual health in the past 12 mo			
Yes	35.0 (28.4-42.2)	46.9 (40.7-65.2)	28.6 (21.1-37.4)
No	65.0 (57.6-71.9)	53.1 (40.7-65.2)	71.4 (62.6-78.9)
Received a question about sexual health from an HCP in the past 5 y			
Yes	2.2 (0.6-5.5)	6.2 (1.8-15.2)	0
No, but needed it	8.2 (4.7-13.2)	12.5 (5.6-23.2)	5.9 (2.4-11.7)
No, but did not need it	85.2 (79.3-90.0)	76.6 (64.3-86.2)	89.9 (83.0-94.7)
Do not know	4.4 (1.9-8.4)	4.7 (1.0-13.1)	4.2 (1.4-9.5)

Abbreviation: HCP, health care provider.

^a^95% CIs were calculated with the Clopper-Pearson exact method.

**Figure 1:**
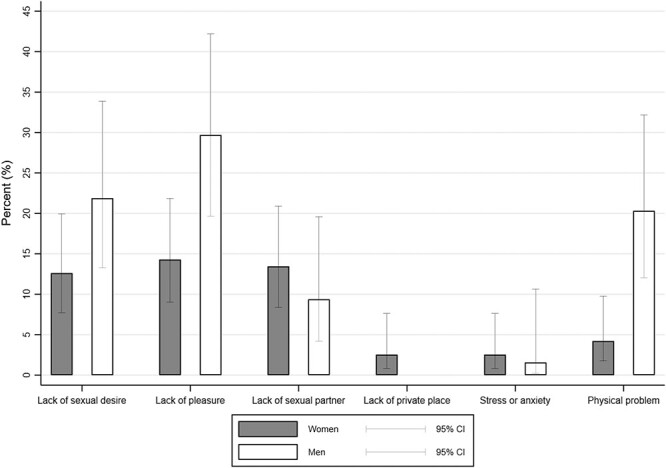
Specific problems related to sexual health reported by female and male participants, expressed as percentages with 95% CIs (*N* = 183). 95% CIs were calculated with the Clopper-Pearson exact method.

Regarding the specific problems related to sexual health, 29.7% (95% CI, 18.9%-42.4%) of men reported a lack of pleasure when having sex; 21.9% (95% CI, 12.5%-40%), a lack of sexual desire; and 20.3% (95% CI, 11.3%-32.2%), a physical problem affecting their sex lives (Figure 1). Among women, 14.3% (95% CI, 8.5%-21.9%) reported a lack of pleasure when having sex; a lack of a sexual partner was the second-most common problem (13.4%; 95% CI, 7.9%-20.9%), whereas 12.6% (95% CI, 7.2%-19.9%) cited a lack of sexual desire and 4.2% (95% CI, 1.4%-9.5%) indicated a physical problem related to sexual health. Last, 2.5% (95% CI, 0.5%-7.2%) of women identified a lack of private space for having sex, while no men reported such a problem. Only the sex difference in having a physical health problem related to sexual health reached statistical significance.

### Multivariable logistic regression for the association between participants’ characteristics and sexual health

Characteristics of the study participants according to their sexual health status are shown in Table S1. In logistic regression, partnered individuals were significantly more likely to be sexually active than those without a partner (aOR, 9.13; 95% CI, 2.53-32.90; Table 3). In addition, having strong social support was positively associated with being sexually satisfied (aOR, 2.96; 95% CI, 1.53-5.74).

**Table 3:** Multivariable binary logistic regression: association between participants’ characteristics and sexual health among the oldest old (≥85 years) in Stockholm County (*N* = 183).

	**In the Past 12 mo, aOR (95% CI)** ^**a**^		
**Characteristic**	**Sexually active**	**Sexually satisfied**	**Having any problem related to sexual health**
Age group, y			
≥90	1 [Reference]	1 [Reference]	1 [Reference]
85-89	1.32 (0.39-4.42)	0.96 (0.48-1.91)	1.97 (0.94-4.13)
Sex			
Women	1 [Reference]	1 [Reference]	1 [Reference]
Men	1.05 (0.35-3.15)	0.68 (0.32-1.42)	1.62 (0.79-3.35)
Partnership status			
Not partnered	1 [Reference]	1 [Reference]	1 [Reference]
Partnered	9.13 (2.53-32.90)	2.01 (0.93-4.36)	1.82 (0.88-3.74)
Having strong social support			
No	1 [Reference]	1 [Reference]	1 [Reference]
Yes	1.44 (0.46-4.45)	2.96 (1.53-5.74)	1.20 (0.61-2.36)
Educational level			
University degree	1 [Reference]	1 [Reference]	1 [Reference]
Nonuniversity degree	1.65 (0.57-4.79)	1.58 (0.81-3.11)	1.15 (0.58-2.31)
Self-perceived health			
Fair to poor	1 [Reference]	1 [Reference]	1 [Reference]
Good	1.90 (0.68-5.34)	1.23 (0.62-2.44)	1.32 (0.67-2.62)
Risk of depression (GDS-4)			
High	1 [Reference]	1 [Reference]	1 [Reference]
Low	6.16 (0.73-52.21)	0.83 (0.37-1.87)	1.33 (0.57-3.06)
Alcohol consumption			
Medium to low	1 [Reference]	1 [Reference]	1 [Reference]
Medium to high	1.73 (0.60-4.98)	0.62 (0.31-1.26)	1.79 (0.90-3.52)
Smoking history			
Smoked	1 [Reference]	1 [Reference]	1 [Reference]
Never smoked	1.52 (0.53-4.33)	1.20 (0.61-2.36)	0.68 (0.35-1.33)
McFadden’s pseudo *R*^2^, %	23.5	7.4	9.5

Abbreviations: aOR, adjusted odds ratio; GDS-4, 4-item Geriatric Depression Scale.

^a^Adjusted for age group, sex, partnership status, having strong social support, educational level, self-perceived health, risk of depression, alcohol consumption, and smoking history, when applicable.

### Post hoc analysis

In logistic regression, being sexually active during the past year was significantly associated with reporting sexual satisfaction, after adjustments for participants’ characteristics (aOR, 7.13; 95% CI, 1.46-34.85). Strong social support was also positively associated with sexual satisfaction, after adjustment for sexual activity and participants’ characteristics (aOR, 3.03; 95% CI, 1.54-5.99).

Table S2 shows participants’ characteristics in relation to being sexually nonsatisfied, sexually nonactive and satisfied, or sexually active and satisfied. In multinomial logistic regression, participants with strong social support were more likely to be sexually satisfied and nonsexually active than sexually nonsatisfied (aOR, 2.98; 95% CI, 1.50-5.93; Table 4). Additionally, having a partner was a significant predictor of being sexually active and satisfied as compared with being sexually nonsatisfied (aOR, 16.21; 95% CI, 3.54-74.31).

**Table 4:** Multinomial logistic regression analysis: association between participants’ characteristics and being sexually active/nonactive and sexually satisfied/nonsatisfied (*N* = 183).

	**aOR (95% CI)** ^**a**^	
**Characteristic**	**Sexually nonactive/satisfied vs nonsatisfied**	**Sexually active/satisfied vs nonsatisfied**
Sex		
Men	1 [Reference]	1 [Reference]
Women	1.54 (0.71-3.33)	1.31 (0.38-4.48)
Age, y		
85-89	1 [Reference]	1 [Reference]
≥90	1.08 (0.53-2.20)	1.01 (0.26-3.81)
Partnership status		
Not partnered	1 [Reference]	1 [Reference]
Partnered	1.35 (0.60-3.02)	16.21 (3.54-74.31)
Having strong social support		
No	1 [Reference]	1 [Reference]
Yes	2.98 (1.50-5.93)	3.32 (0.93-11.89)
Educational level		
University degree	1 [Reference]	1 [Reference]
Nonuniversity degree	1.36 (0.68-2.75)	3.06 (0.88-10.61)
Self-perceived health		
Good	1 [Reference]	1 [Reference]
Poor to fair	1.10 (0.54-2.23)	2.00 (0.63-6.37)
Risk of depression (GDS-4)		
Low	1 [Reference]	1 [Reference]
High	1.37 (0.61-3.01)	0.21 (0.02-2.02)
Alcohol consumption		
Medium to high	1 [Reference]	1 [Reference]
Medium to low	1.75 (0.85-3.61)	1.07 (0.32-3.55)
Smoking history		
Smoked	1 [Reference]	1 [Reference]
Never smoked	1.15 (0.57-2.31)	1.20 (0.37-3.85)
McFadden’s pseudo *R*^2^, %	13.8	

Abbreviations: aOR, adjusted odds ratio; GDS-4, 4-item Geriatric Depression Scale.

^a^Adjusted for age group, sex, partnership status, having strong social support, educational level, self-perceived health, risk of depression, alcohol consumption, and smoking history, when applicable.

## Discussion

We found that 12% of participants were sexually active during the past year and that having a partner was positively associated with sexual activity. Most (63.9%) reported being satisfied with their sexual lives, and having strong social support was positively associated with sexual satisfaction. Approximately one-third experienced a problem related to sexual health during the past year, and men were more likely than women to report a physical health problem affecting their sexual lives. Only 2.2% had received a question on sexual health from an HCP during the past 5 years, and 8.2% reported an unaddressed need for such an inquiry. Yet, most did not report such a need.

Our findings align with 2 previous studies finding that 10% of individuals older than 90 years were sexually active, as well as 31% of men and 14% of women aged ≥80 years.^32^^,^^33^ Our results build on the existing literature, which consistently demonstrates a decline in sexual activity with increasing age.^18^^,^^32–34^ Additionally, we found that partnered individuals were more likely to report sexual activity, which corroborates previous findings that being married or having a partner positively affects sexual activity in older people.^17^^,^^18^^,^^32^^,^^35^ It could be hypothesized that this finding occurred because one’s partner is also likely to be one’s sexual partner, although this was not addressed in our study and we could not exclude other explanations.

In crude analysis, men were more likely than women to report a physical problem affecting their sexual health—an effect that was attenuated in adjusted analysis. According to the Massachusetts Male Aging Study and the European Male Aging Study, the prevalence of erectile dysfunction is positively associated with increasing age, estimated to be as high as 64% among men aged ≥70 years, whereas data from 4 European countries revealed that a large proportion of women among those aged 60 to 75 years experience vaginal dryness (47.7%) and pain during sex (23.5%).^27^^,^^28^

Previous studies have highlighted the important role of social support in the lives and health status of older individuals.^36–41^ Our study contributes to the existing knowledge by revealing a positive association between having strong social support and experiencing sexual satisfaction among the oldest old. Sexual satisfaction, being linked to overall life satisfaction, may be an additional benefit of having a supportive social network, contributing to an older individual’s sense of fulfilment and well-being.^42^^,^^43^

Interestingly, 53% of study participants reported being sexually satisfied and nonactive. Having strong social support was associated with a higher likelihood of being sexually satisfied and nonactive, as compared with being sexually nonsatisfied. There was also a positive association between having strong social support and the likelihood of being sexually active and satisfied, as compared with being sexually nonsatisfied. However, this latter association was not statistically significant, possibly due to the lack of statistical power as there were few study participants who were sexually active and satisfied. Furthermore, as discussed previously, strong social support was associated with a higher likelihood of sexual satisfaction even after adjustments for sexual activity and participants’ characteristics. These findings may offer some insight into the apparent discrepancy in literature between the decline in sexual activity with increasing age and the relatively stable rates of sexual satisfaction in older people.^17^^,^^18^ It could be hypothesized that participants with a strong social supportive network may tend to replace the importance of sexual activity for sexual satisfaction with the security and intimacy provided by their social networks.

Among older people, problems related to sexual health have been associated with a higher risk of other adverse health outcomes.^44^ Although problems related to sexual health were reported by 35% of participants, only 2.2% had their sexual health addressed by an HCP, while 12.5% of men and 5.9% of women expressed such a need that was not addressed. These findings might indicate unmet health care needs regarding the sexual health of the oldest old, especially in males. Nevertheless, 85.2% of the participants did not express a need for being asked about their sexual health by an HCP. Yet, older people might internalize social stereotypes on the asexual older individual, which may hinder them from perceiving their problems related to sexual health as something worth or acceptable to discuss with HCPs.^45^

Strengths of our study include the population-based design, information on sexual health of an underresearched population group, and that we were able to control our analyses for important confounders. However, there are limitations. The generalizability of our results might be limited due to the small sample size, which is a major limitation. Yet, the only prior study from Sweden that reported on sexual activity among the oldest old in a medium-sized Swedish town also found that 10% to 25% of the participants in this age group were sexually active, which corroborates and thus strengthens our findings.^32^ Moreover, our findings might not be readily generalized to countries outside Sweden or to nonurban settings. Another limitation is that the relatively small sample size required us to categorize variables into binary groups to maintain statistical power, thereby losing valuable information from the individual categories, which might explain the low explanatory power in a few models. We also lacked detailed information, such as the frequency or nature of sexual activity. Last, a major limitation of our study is that, due to the cross-sectional data and lack of causal inference design, we cannot make any causal interpretation of the observed associations between participants’ characteristics and sexual health.

## Conclusion

This study found that about a 10th of the oldest old in Stockholm County, Sweden, were sexually active during the past year, whereas about two-thirds were satisfied with their sexual lives. Approximately a third had experienced a problem related to sexual health during the past year, and men were more likely than women to report a physical health problem affecting their sex lives. A tiny proportion of men and no women received inquiries from an HCP regarding their sexual health. However, most did not express a need to discuss their sexual health with an HCP either. These findings nevertheless indicate a need for future studies to explore the potential of unmet health care needs in relation to sexual health among the oldest old, as well as potential barriers in communicating sexual health needs in this population.

## Supplementary Material

Appendix_clean_qfae022

## Data Availability

All data supporting the findings of this study are available within the article and its supplementary material.
