# Supplementary material for: Sexual health among the oldest old: a population-based study among people aged 85 years and older in Stockholm, Sweden
Source: Sex Med. 2024 Apr 5;12(2):qfae022. doi: 10.1093/sexmed/qfae022 (PMC10997412; doi:10.1093/sexmed/qfae022)
Supplement: Appendix_clean_qfae022 [file appendix_clean_qfae022.docx]

**Appendix**

**Table 1.** Sociodemographic- and health-related characteristics of the study participants (n=183) by sexual health status.

|  | **Sexually active during**  **the past 12 months** | | | **Sexually satisfied during**  **the past 12 months** | | | **Any problem related to sexual health during the past 12 months** | | |
| --- | --- | --- | --- | --- | --- | --- | --- | --- | --- |
| **Characteristic n (%)**  **Sex**  Men  Women  **Age**  85-89 years  ≥ 90 years  **Partnership status**  Partnered  Not partnered  **Strong social support**  Yes  No  **Educational level**  University degree  Non university degree  **Self-perceived health**  Good  Poor to fair  **GDS-4 ***  High risk for depression  Low risk for depression  **Alcohol consumption**  Medium to high  Medium to low  **Smoking history**  Having smoked  Never having smoked | **Yes**  (n=22)  12 (54.5)  10 (45.5)  17 (77.3)  5 (22.7)  18 (81.8)  4 (18.2)  16 (72.7)  6 (27.3)  7 (31.8)  15 (68.2)  12 (54.5)  10 (45.5)  1 (4.5)  21 (95.5)  14 (63.6)  8 (36.4)  8 (36.4)  14 (63.6) | **No/Don’t know**  (n=161)  52 (32.3)  109 (67.7)  106 (65.8)  55 (34.2)  50 (31.1)  111 (68.9)  96 (59.6)  65 (40.4)  56 (34.8)  105 (65.2)  61 (37.9)  100 (62.1)  39 (24.2)  122 (75.8)  60 (37.3)  101 (62.7)  66 (41.0)  95 (59.0) | **p-value**  0.040  0.28  <0.001  0.24  0.78  0.13  0.036  0.018  0.68 | **Yes**  (n=117)  38 (32.5)  79 (67.5)  78 (66.7)  39 (33.3)  47 (40.2)  70 (59.8)  82 (70.1)  35 (29.9)  37 (31.6)  80 (68.4)  49 (41.9)  68 (58.1)  26 (22.2)  91 (77.8)  45 (38.5)  72 (61.5)  45 (38.5)  72 (61.5) | **No/Don’t know**  (n=66)  26 (39.4)  40 (60.6)  45 (68.2)  21 (31.8)  21 (31.8)  45 (68.2)  30 (45.5)  36 (54.5)  26 (39.4)  40 (60.6)  24 (36.4)  42 (63.6)  14 (21.2)  52 (78.8)  29 (43.9)  37 (56.1)  29 (43.9)  37 (56.1) | **p-value**  0.35  0.83  0.26  0.001  0.29  0.46  0.87  0.47  0.47 | **Yes**  (n=64)  30 (46.9)  34 (53.1)  50 (78.1)  14 (21.9)  33 (51.6)  31 (48.4)  42 (65.6)  22 (34.4)  22 (34.4)  42 (65.6)  29 (45.3)  35 (54.7)  11 (17.2)  53 (82.2)  35 (54.7)  29 (45.3)  32 (50.0)  32 (50.0) | **No**  (n=119)  34 (28.6)  85 (71.4)  73 (61.3)  46 (38.7)  35 (29.4)  84 (70.6)  70 (58.8)  49 (41.2)  41 (34.5)  78 (65.5)  44 (37.0)  75 (63.0)  29 (24.4)  90 (75.6)  39 (32.8)  80 (67.2)  42 (35.3)  77 (64.7) | **p-value**  0.013  0.021  0.003  0.37  0.99  0.27  0.26  0.004  0.053 |

Chi-squared test p-values. All cells have expected counts of at least 3. * GDS-4 stands for 4-item geriatric depression scale.

**Table 2.** Sociodemographic- and health-related characteristics of the study participants (n=183) according to being either i) sexually non-satisfied, or ii) sexually non-active and sexually satisfied, or iii) sexually active and sexually satisfied.

|  | **Sexually satisfied and sexually non-active**  **VS sexually non-satisfied *** | | | **sexually satisfied and sexually active**  **VS sexually non-satisfied *** | | |
| --- | --- | --- | --- | --- | --- | --- |
| **Characteristic n (%)**  **Total**  **Sex**  Men  Women  **Age**  85-89 years  ≥ 90 years  **Partnership status**  Partnered  Not partnered  **Having strong social support**  Yes  No  **Educational level**  University degree  Non university degree  **Self-perceived health**  Good  Poor to fair  **GDS-4 ****  High risk for depression  Low risk for depression  **Alcohol consumption**  Medium to high  Medium to low  **Smoking history**  Having smoked  Never having smoked | **Sexually satisfied and sexually non-active**  (n=97)  27 (27.8)  70 (72.2)  63 (64.9)  34 (35.1)  30 (69.1)  67 (30.9)  67 (69.1)  30 (30.9)  32 (33.0)  65 (67.0)  38 (39.2)  59 (60.8)  25 (25.8)  72 (74.2)  33 (34.0)  64 (66.0)  37 (38.1)  60 (61.9) | **Sexually non-satisfied**  (n=66)  26 (39.4)  40 (60.6)  45 (68.2)  21 (31.8)  21 (31.8)  45 (68.2)  30 (45.5)  36 (54.5)  26 (39.4)  40 (60.6)  24 (36.4)  42 (63.6)  14 (78.8)  52 (21.2)  29 (43.9)  37 (56.1)  29 (43.9)  37 (56.1) | **p-value**  0.12  0.67  0.90  0.003  0.40  0.72  0.50  0.20  0.46 | **Sexually satisfied and active**  (n=20)  11 (55.0)  9 (45.0)  15 (75.0)  5 (25.0)  17 (85.0)  3 (15.0)  15 (75.0)  5 (25.0)  5 (25.0)  15 (75.0)  11 (55.0)  9 (45.0)  1 (5.0)  19 (95.0)  12 (60.0)  8 (40.0)  8 (40.0)  12 (60.0) | **Sexually non-satisfied**  (n=66)  26 (39.4)  40 (60.6)  45 (68.2)  21 (31.8)  21 (31.8)  45 (68.2)  30 (45.5)  36 (54.5)  26 (39.4)  40 (60.6)  24 (36.4)  42 (63.6)  14 (78.8)  52 (21.2)  29 (43.9)  37 (56.1)  29 (43.9)  37 (56.1) | **p-value**  0.047  0.56  <0.001  0.020  0.24  0.14  0.094  0.21  0.76 |

Chi-squared test p-values. All cells have expected counts of at least 3. *During the past 12 months. ** GDS-4 stands for 4-item geriatric depression scale.
